# Lung Ultrasound Reduces Chest X-rays in Postoperative Care after Thoracic Surgery: Is There a Role for Artificial Intelligence?—Systematic Review

**DOI:** 10.3390/diagnostics13182995

**Published:** 2023-09-19

**Authors:** Marek Malík, Anton Dzian, Martin Števík, Štefánia Vetešková, Abdulla Al Hakim, Maroš Hliboký, Ján Magyar, Michal Kolárik, Marek Bundzel, František Babič

**Affiliations:** 1 Department of Thoracic Surgery, Jessenius Faculty of Medicine in Martin, Comenius University in Bratislava and University Hospital in Martin, Kollárova 4248/2, 036 59 Martin, Slovakia; 2 Radiology Department, Jessenius Faculty of Medicine in Martin, Comenius University in Bratislava and University Hospital in Martin, Kollárova 4248/2, 036 59 Martin, Slovakia; 3 Department of Cybernetics and Artificial Intelligence, Faculty of Electrical Engineering and Informatics, Technical University of Košice, Letná 9, 040 01 Košice, Slovakia

**Keywords:** lung ultrasound, artificial intelligence, deep learning, thoracic surgery, postoperative management

## Abstract

Background: Chest X-ray (CXR) remains the standard imaging modality in postoperative care after non-cardiac thoracic surgery. Lung ultrasound (LUS) showed promising results in CXR reduction. The aim of this review was to identify areas where the evaluation of LUS videos by artificial intelligence could improve the implementation of LUS in thoracic surgery. Methods: A literature review of the replacement of the CXR by LUS after thoracic surgery and the evaluation of LUS videos by artificial intelligence after thoracic surgery was conducted in Medline. Results: Here, eight out of 10 reviewed studies evaluating LUS in CXR reduction showed that LUS can reduce CXR without a negative impact on patient outcome after thoracic surgery. No studies on the evaluation of LUS signs by artificial intelligence after thoracic surgery were found. Conclusion: LUS can reduce CXR after thoracic surgery. We presume that artificial intelligence could help increase the LUS accuracy, objectify the LUS findings, shorten the learning curve, and decrease the number of inconclusive results. To confirm this assumption, clinical trials are necessary. This research is funded by the Slovak Research and Development Agency, grant number APVV 20-0232.

## 1. Introduction

The importance of lung ultrasound (LUS) has grown from the 1980s to the 20th century. In the beginning, LUS did not look useful for chest and lung evaluations due to the ribs and lungs being two environments that represent an obstacle for ultrasound waves. The identification of LUS signs [1,2,3,4,5,6] led to its increased importance, especially in intensive care medicine, and the creation of clinical protocols [7,8] and complex guidelines in this field [9,10]. Nowadays, lung ultrasound plays an important role in various medical specialties like pneumology, internal medicine, traumatology, cardiothoracic surgery, etc. [11,12,13,14].

In postoperative care after non-cardiac thoracic surgery, especially in the last decade, increased interest in LUS was also recorded. CXR remains the standard imaging modality in the monitoring of surgically induced pneumothorax (PTX), pleural effusion (PE), and postoperative complications in this field. Growing evidence has shown the potential for LUS to reduce chest X-rays (CXR) in postoperative care after non-cardiac thoracic surgery and in chest tube management [15,16,17,18,19,20,21,22,23,24,25,26,27]. The optimal management of chest tubes and their removal after non-cardiac thoracic surgery and the role of imaging modalities in the detection of PTX, PE, and other conditions remain unclear [28,29,30,31,32]. Standardized protocols among thoracic surgery departments are missing, and the strategy varies from daily routine CXR to the omission of any imaging modality, especially thanks to the introduction of modern digital drainage systems [33,34]. Several trials studied the role of CXR in postoperative care after non-cardiac thoracic surgery with the goal of decreasing the CXR numbers [35,36]. In 2019, guidelines for enhanced recovery after lung surgery were published by the Enhanced Recovery After Surgery (ERAS^®^) Society and by the European Society of Thoracic Surgeons (ESTS), but recommendations regarding the standardized use of imaging modalities in postoperative care are missing [37].

As mentioned above, several trials have shown that LUS could represent an alternative approach to decrease the CXR in the postoperative period after non-cardiac thoracic surgery and thus lower the radiation [38]. In 2012, Goudie et al. published the first relevant clinical trial conducted in thoracic surgery regarding the use of LUS in postoperative care [15]. Newer trials in this field improved the used methods and LUS protocols. To our best knowledge, the most complex ultrasound protocol in this field was used by Galetin et al. [19] by introducing the Bedside Lung Ultrasound in Emergency (BLUE) protocol [7] in postoperative care after non-cardiac thoracic surgery.

Along with the growing importance of LUS in clinical practice, the last decade also brought a growing interest in the evaluation of radiological pictures by artificial intelligence (AI). AI has allowed us to evaluate information from images that is invisible to the human eye. Evaluation of lung nodules on computed tomography (CT) scans using AI and the concept of radiomics could serve as a good example [39,40,41,42].

LUS has shown to be an ideal imaging modality for the quick assessment of COVID-19 patients with the ability to “predict the clinical course and outcome” [43]. The COVID-19 pandemic has allowed a broader expansion of AI methods in LUS evaluation [44,45,46]. It was confirmed that AI “can distinguish similar appearing LUS pathology, including COVID-19, that cannot be distinguished by humans. The performance gap between humans and the model suggests that subvisible biomarkers within ultrasound images could exist” [47].

Our initial experience with the evaluation of LUS videos from patients after non-cardiac thoracic surgery using deep learning and AI [48,49] encouraged us to perform a literature review with two aims. The first aim was to review the state of the art regarding the use of LUS in postoperative care and chest tube management after non-cardiac thoracic surgery to assess whether lung ultrasound could replace CXR. Also, we want to identify possible areas where AI could help in the broader implementation of LUS in postoperative care after non-cardiac thoracic surgery. The second aim was to review the literature regarding the use of AI in the evaluation of LUS videos of patients in the postoperative period after non-cardiac thoracic surgery. Nowadays, the existing evidence on AI use in LUS evaluation is mostly from COVID-19 patients. Despite this fact, we found it reasonable to evaluate AI use in LUS in thoracic surgery for several reasons. Patients after thoracic surgery represent a specific group with specific clinical conditions and complications. Although the lung and pleural pathologies and the LUS signs will be the same, the prevalence of the evaluated pathologies and the prevalence of the evaluated LUS signs may differ. The main goal of the COVID-19 pandemic was to triage healthy individuals, patients with bacterial pneumonia, and patients with COVID-19 pneumonia and predict their clinical course and outcome [46]. In patients after non-cardiac thoracic surgery, the main goal is to evaluate surgically induced pneumothorax, pleural effusion, and postoperative complications.

## 2. Materials and Methods

Based on the aims of this review and according to Preferred Reporting Items for Systemic Reviews and Meta-Analyses (PRISMA) guidelines [50], two searches in Medline (Pubmed) were performed on 22 June 2023.

The first search in Medline was performed to identify relevant articles regarding the use of LUS in postoperative care, especially in chest tube management, after non-cardiac thoracic surgery in adults. For this search, the following keywords and their combinations were used: “lung/chest ultrasound/ultrasonography/sonography” AND “thoracic surgery/lung surgery/lung resection”. Studies reporting the use of LUS in postoperative period after all types of non-cardiac thoracic procedures in adults were included. For evaluation of the LUS as a tool in chest tube management, the LUS diagnostic of PTX was a condition to include the study into our review. Studies on patients undergoing cardiac surgery, non-thoracic surgery, studies on pediatric patients (under 18 years), studies without LUS evaluation of the PTX, animal studies, editorials, letter to editor, reviews, etc. were excluded. Reference lists of included articles were manually searched for relevant studies.

The second search in Medline was performed to identify relevant articles regarding the evaluation of LUS videos from the postoperative period after non-cardiac thoracic surgery with the help of AI methods. For the second search, combinations of the following keywords were used: “thoracic surgery/lung surgery/lung resection” AND “ultrasound/ultrasonography/sonography” AND “artificial intelligence/deep learning”. Studies reporting the use of the AI method in LUS videos evaluation in postoperative care after all types of non-cardiac thoracic procedures in adults were included. Studies on patients undergoing cardiac surgery, non-thoracic surgery, studies on pediatric patients (under 18 years) and animal studies were excluded.

Our research is funded by the Slovak Research and Development Agency, grant number APVV 20-0232, and it was approved by the Ethical Committee of Jessenius Faculty of Medicine in Martin, Slovakia (No. EK 44/2021). The research is conducted in accordance with the Declaration of Helsinki. Written informed consent was obtained from all subjects involved in the study prior to examination.

## 3. Results

### 3.1. LUS in Postoperative Care after Non-Cardiac Thoracic Surgery

**Figure 1:**
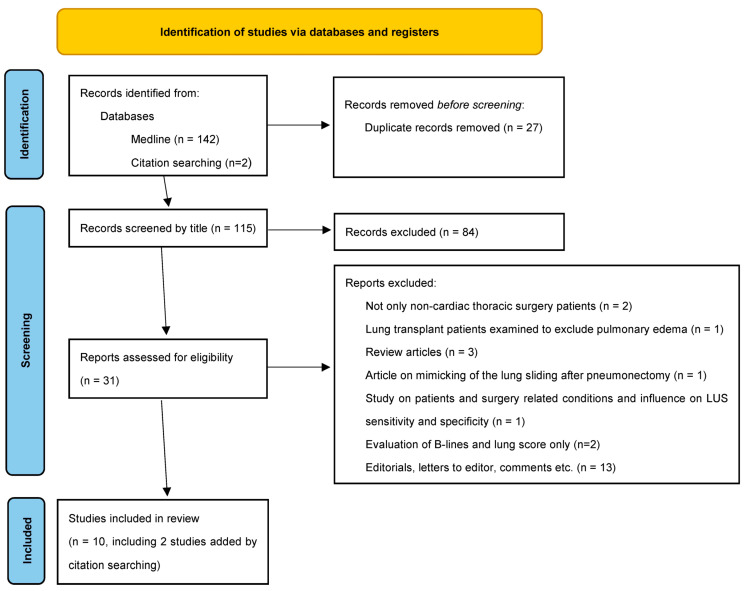
PRISMA flow diagram for identification of the trials on the use of lung ultrasound in postoperative care after non-cardiac thoracic surgery [50].

The Medline (Pubmed) search for keywords as mentioned in the methods identified 142 articles about the use of LUS in postoperative care and chest tube management after non-cardiac thoracic surgery. Of these, 27 duplicate records were removed, 115 articles were screened by title, and 84 of them were excluded as irrelevant. Overall, 31 articles were screened by abstract for eligibility and 23 of them were excluded as irrelevant due to exclusion criteria. A total of 8 trials met the inclusion criteria [16,17,18,19,22,23,25,26]. Reference lists of these relevant articles were analyzed, and two more studies [15,20] were included in our review. Overall, 10 studies were analyzed in this review (Figure 1, Table 1).

#### 3.1.1. Basic Characteristics of the Reviewed Trials

The aim of the reviewed trials was to evaluate the role of LUS and CXR in the postoperative period and chest tube management after non-cardiac thoracic surgery. Nine of these trials have evaluated whether it is possible to replace or reduce the CXR by LUS. Smargiassi et al. [18] evaluated the complementary role of these two imaging modalities after non-cardiac thoracic surgery based on the suspected complication and physical examination.

#### 3.1.2. Summary of the Reviewed Trials Methods

All studies were observational single-center trials. Smargiassi et al. [18] enrolled patients in their study retrospectively; the rest were prospective trials. A total of 919 patients have undergone 1926 LUS and CXR examination. These numbers are uncertain, because from several research teams, more than one article was included in this review, and according to patients’ characteristics, it is possible that certain patients were included in more than one study.

In Goudie et al. [15] and Malík et al. [22], patients after the whole spectrum of non-cardiac thoracic procedures were included, including procedures without lung resections. Dzian et al. [23] and Messina et al. [26] evaluated patients after major lung resections only; lobectomy was the most common procedure. In Smargiassi et al. [18], thoracotomies, mini/invasive procedures, and robotic thymectomies were performed, but the details regarding surgical procedures were not available. The rest of the trials enrolled patients where the postoperative presence of air leakage was expected: i.e., both anatomical and wedge lung resections [16,17]; lung resections (both anatomical and wedge) and/or chest wall resections [19,20]; lung resections (both anatomical and wedge) and decortications [20,26].

The most common exclusion criterium was the pneumonectomy due to empty pleural space without the possibility of LUS signs evaluation. Other reported exclusion criteria were subcutaneous emphysema [25,26] and wound dressing as an obstacle for LUS examination [26]. Inconclusive LUS results were excluded from statistical evaluation in several trials [20,22,23]. The most restrictive exclusion criteria were used in the study of Patella et al. Patients with no air leak, with subcutaneous emphysema, with the presence of severe chronic obstructive lung disease, patients after chest wall or diaphragm resection, with respiratory distress, pleurodesis and pleurectomy were not included in the study [17].

In all trials, LUS and CXR were performed. Mostly, CXR served as a special case of imperfect reference test with 100% specificity [19]. LUS was performed every time CXR was performed, but the strategy varied from postoperative examination [16,18], examination after chest tube removal [18,19], combination of postoperative examination and examination prior to chest tube removal [22,23], postoperative examination and examination after chest tube removal [20], postoperative examination and examinations prior and also after chest tube removal [25] and daily routine examination until chest tube removal [26].

The LUS was performed to detect PTX and lung re-expansion only [17,19,20,25]; or to detect PTX and PE [15,22,23]; or evaluation of PTX, PE, and lung consolidations [26]; or detection of PTX, PE, lung consolidations, subcutaneous emphysema and diaphragm position [16,18]. Evaluated LUS signs varied from detection of lung sliding only [17] to the use of complex protocols with additional signs [19,20].

All LUS examiners were blinded to CXR results. Examiners have different experiences in LUS among studies. In Goudie et al. [15], the examiners, thoracic surgeons, had limited experience with LUS. In the rest of the studies, the authors declared skilled LUS examiners from various medical fields (thoracic surgeons [16,18,19,20,22,23,25,26], pneumologists [16,18] and radiologists [17]).

Statistical evaluations differ among performed trials. Several trials used diagnostic accuracy tests [15,17,22,25]. Some studies have used the statistical methodology for the use of CXR as an imperfect reference [19,20,23]. Several studies evaluated the agreement between LUS and CXR [18,22,23,26] or therapeutical agreement based on LUS and CXR [19,20,25,26]. Some trials evaluated the exhaustiveness of LUS for clinical decision making [16] or the percentage of saved CXR [16,17,22,23].

#### 3.1.3. Summary of the Reviewed Trials Results

All performed trials evaluated PTX. As shown in Table 1, the sensitivity of LUS compared to CXR varies from 21.2% [15] to 86% [25], while the specificity varies from 85% [19] to 100% [25], the positive predictive value varies from 52.7% [15] to 94% [25], and the negative predictive value varies from 62.2% [23] to 100% [17]. Percentage of agreement varies from 72% [26] to 92.3% [23] and Cohen’s Kappa varies from 39.7% to 77.5% [23]. The sensitivity of LUS for PTX, when compared to CXR, increases with the PTX size. For pneumothoraxes with an apex-to-cupula distance of 1, 2 and 3 cm, the LUS sensitivity was 43%, 56% and 100%, respectively, in Galetin et al. [19], and it was 78%, 86%, and 100%, respectively, in Messina et al. [25]. Several trials showed that no clinically important PTX (apex-to-cupula distance PTX > 3 cm according to the American College of Chest Physicians Delphi Consensus Statement on pneumothorax [51]) was missed by LUS [17,19,20,22,23,25]. In Smargiassi et al., no PTX was missed by LUS [18]. For PTX, four trials [19,20,25,26] evaluated therapeutical agreement between LUS and CXR-based clinical decision with agreement from 94% [26] to 97% [19,25].

Five trials evaluated the PE. The sensitivity of LUS compared to CXR varied from 32.6% [23] to 83.1% [15], the specificity varied from 59.3% [15]–92.6% [22], the positive predictive value varied from 33.1% [23] to 88.3% [23], and the negative predictive value varied from 12.2% [23] to 99% [23]. The percentage of agreement varied from 38% [26] to 88.1 [23], and Cohen’s Kappa varied from 39% [16] to 61.1% [23]. Only Jakobson et al. assessed the therapeutic agreement between LUS and CXR-based clinical decisions and found 80% agreement.

In the reviewed trials, no diagnostic accuracy test was performed for other pathologies than PTX and PE. Agreement between LUS and CXR was studied for lung consolidations, subcutaneous emphysema and for diaphragm position.

For lung consolidations, the percentage of agreement between LUS and CXR was 50% and Cohen’s Kappa was 6% in Smargiassi et al. [18], and there was 100% diagnostic and 100% therapeutic agreement in Jakobson et al. [26].

In Smargiassi et al., the percentage of agreement between LUS and CXR for subcutaneous emphysema was 58%, while Cohen’s Kappa was 21%, and for diaphragm position, the percentage of agreement was 91%, and Cohen’s Kappa was 70% [18].

In several trials, the evaluation of exhaustiveness of LUS results was performed to find out how many CXR it would be possible to save. In Chiappetta et al., 67% of LUS in general and 85% of LUS after mini-invasive procedures were exhaustive enough to make clinical decisions based on LUS, and thus, it was possible to omit CXR [16]. In Dzian et al., 77% of CXR after major lung resections could be saved [23]. In a trial conducted by Patella et al., 86% of CXR after chest tube removal was not necessary thanks to LUS [17]. Using LUS as the first imaging modality in postoperative care, it would be possible to save 61.6% of CXR in the case of physiological finding according to Malík et al. [22].

#### 3.1.4. Possible Areas Where Evaluation of LUS Videos Using AI Could Be Helpful

Limitations and discussed topics were summarized to identify the possible areas where an evaluation of LUS videos with AI could be helpful. The main limitation for lung ultrasound in all performed trials was severe subcutaneous emphysema. Wound dressing was not a major limitation. In Malík et al. [22] and Dzian et al. [23], the missing lung points in case of abolished lung sliding have led to significant numbers of inconclusive LUS results. Investigator‘s education and experience is an often-discussed topic. The subjectivity of LUS findings is discussed as well.

#### 3.1.5. Summary of the Reviewed Trials Conclusions

Eight out of ten reviewed trials concluded that LUS used to replace or reduce the CXR in postoperative care after non-cardiac thoracic surgery. Smargiassi et al. evaluated the complementary role of these two modalities, but these authors also concluded that LUS is able to detect important findings that could be missed by CXR [18]. Only Goudie et al. did not recommend replacing CXR by LUS in this field [15].

### 3.2. AI in LUS Images Evaluation in Postoperative Care after Non-Cardiac Thoracic Surgery

**Figure 2:**
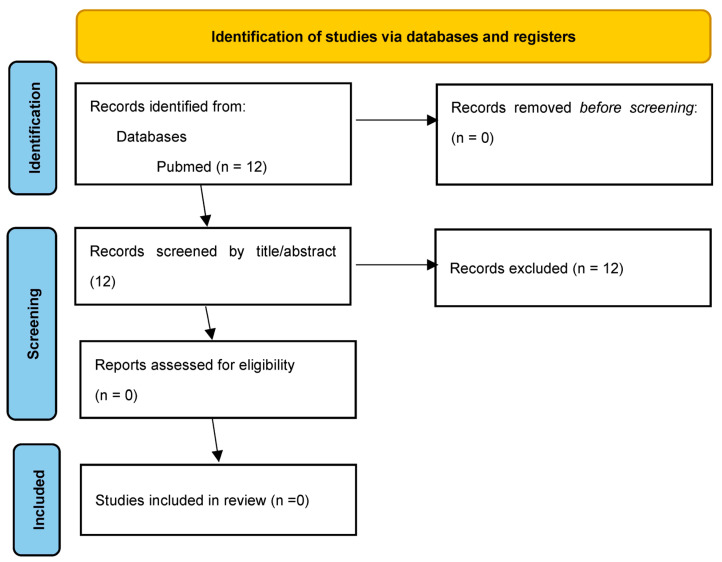
PRISMA flow diagram for identification of articles regarding the use of artificial intelligence in lung ultrasound videos evaluation in postoperative care after non-cardiac thoracic surgery [50].

The Medline (PubMed) search for keywords as mentioned in the Material and Methods section identified 12 articles. No duplicates were identified. All articles were screened by title and abstract, and all of them were excluded as irrelevant (Figure 2). Due to our best knowledge and the performed literature review, there are no studies evaluating the possible role of AI in the detection of LUS signs in postoperative care after non-cardiac thoracic surgery.

## 4. Discussion

### 4.1. LUS in Postoperative Care after Non-Cardiac Thoracic Surgery

Eight out of ten reviewed clinical trials clearly showed that LUS can significantly reduce CXR in postoperative care and in chest tube management after non-cardiac thoracic surgery without having a negative impact on the decision-making process and patient outcome [16,18,19,20,22,23,25,26]. The aim of Smargiassi et al. was to assess the complementary role of LUS and CXR after non-cardiac thoracic surgery [17]. The author of the Goudie et al. trial [15] concluded that LUS “does not have enough accuracy to replace CXRs”. LUS methods and investigators’ experience could have a major impact on the results of Goudie et al. trial.

According to the heterogeneity of the methods and results of the reviewed trials, it is not possible to perform a consistent meta-analysis. There are significant differences in the inclusion and exclusion criteria of the patients and in the performed thoracic procedures, as shown in Table 1. An important point for the heterogeneity of these trials is the missing standard among thoracic surgery departments regarding the use of drainage systems, postoperative patient evaluation, cut-off points for postoperative air leakage and the mount of fluid per 24 h for chest tube removal indications and standards regarding the use of imaging modality [28,29,30,31,32]. Recommendations regarding the use of imaging modality after thoracic surgery in the guidelines for enhanced recovery after lung surgery are missing [37]. The differences in postoperative care in reviewed trials confirmed this fact. The CXR strategy varied from an on-demand approach to daily routine CXRs (Table 1). When chest tube removal is indicated, some departments performed CXR prior to its removal [22], several departments performed CXR after its removal [17,19] and one trial reported CXR prior to and after chest tube removal [26]. An interesting approach was presented by Lesser et al. In their study, they evaluated the non-use of chest tubes after minor uncomplicated lung resections. They showed a decreased CXR number after lung biopsy by video-assisted thoracoscopic surgery without a chest tube insertion when compared with the group with chest tube insertion. In the no chest tube group, pneumothorax and bleeding were ruled out by repetitive bedside LUS on the day of surgery. The authors concluded that CXR can be completely omitted in the case of ubiquitous lung sliding presence on LUS [52].

Standardization of the postoperative care according to evidence-based medicine principles regarding the used imaging modalities could be helpful for future trials in this field and for their better comparability.

In contrast with the high accuracy of LUS for detected conditions from various medical fields, especially from intensive care and traumatology, the trials from thoracic surgery did not confirm these results. [11,12,13,14]. Goudie et al. performed the first trial in the field of non-cardiac thoracic surgery with PTX sensitivity 21.2% [15]. It was assumed that unexperienced investigators and an insufficient LUS methodology could have a major negative impact on the results of this trial. In this trial, the investigators had only initial experience. PTX was concluded in case of missing lung sliding and missing B-lines, and the presence of a lung point was not investigated [4]. Newer trials have reflected this fact in their study design. More LUS signs were added to PTX diagnosis [22,23]. In several trials, lung pulse and M-mode signs were introduced to LUS methods [25,26]. Several trials introduced BLUE protocol in postoperative care after thoracic surgery [19,20]. In the majority of these trials, LUS was performed by experienced investigators. Adding more ultrasound signs to LUS protocol increases accuracy [19,20,25,26] and decreases the number of inconclusive results. The absence of a lung point in case of abolished lung sliding was a major cause of inconclusive results in some trials with a simplified LUS algorithm for PTX diagnosis [22,23]. This could be solved by adding additional LUS signs. Galetin et al. [19] have reported that lung pulse [5] was the most important LUS sign to exclude PTX. Also, the investigation of lung consolidation could improve the results [16,18,19,20]. Evaluating PTX and PE only assesses the LUS as potential imaging modality for chest tube management [15,17,22,23]. Adding more ultrasound signs to used diagnostic protocols or the use of complex LUS protocols showed the potential of LUS to be a complex tool not only for chest tube management and decision making regarding its removal but for the complex monitoring of postoperative complications [16,18,19,20,26]. On the other hand, complex LUS examination requires a skilled investigator. We assume that artificial intelligence could be helpful and could improve the LUS implementation to daily routine in postoperative care after non-cardiac thoracic surgery by increasing the accuracy and decreasing the number of inconclusive LUS results, especially in the case of limited investigator experience. Initial findings of the use of artificial intelligence in LUS evaluation showed that AI could ”see more” than the human eye on LUS images [47]. If this assumption will be confirmed, the AI could be an important tool for increasing the LUS accuracy.

The improvements in LUS protocols in newer trials in this review have led to better results, but the results did not reach the accuracy from other medical fields. The important issue in the trials from non-cardiac thoracic surgery is the use of CXR as an imperfect reference test with 100% specificity [53]. Unfortunately, the use of the CT as the gold standard would not be ethical in this setting. A meta-analysis by Ding et al. comparing LUS and CXR in PTX diagnostics showed the superiority of LUS (sensitivity 88%, specificity 99%) over CXR (sensitivity 52%, specificity 100%) [54]. The same applies for the superiority of LUS (sensitivity 94%, specificity 98%) over CXR (sensitivity 51%, specificity 91%) in PE diagnostics confirmed in a meta-analysis by Yousefifard et al. [55]. The superiority of LUS over CXR could have a negative impact on the achieved results.

CXR as an imperfect reference test has impacts also on statistics [53]. Different authors have dealt with this fact differently. Some of them have used conventional calculation of sensitivity, specificity, and other statistical values [15,22,25]. Chiappetta et al. and Smargiassi et al. did not use a reference test. They evaluated the exhaustiveness of LUS for clinical decision making [16], and using Cohen’s Kappa, they assessed the agreement between CXR and LUS [18]. Patella et al. did not calculate the sensitivity and specificity. Only positive and negative predictive values were calculated [17]. Galetin et al. [19] highlighted the use of a statistical method that reflects the use of the CXR as an imperfect reference test with 100% specificity in diagnostic accuracy tests [19,53]. In their trials [19,20], the method of Staquet et al. was used [56]. Dzian et al. [23] used thestatistical method of Emerson et al. [57]. There is an issue with both statistical methods [56,57]. For the calculation of diagnostic values, the sensitivity of the gold standard, CT here, for evaluated pathologies is required. The sensitivity of the CT for PTX and PE was not evaluated in postoperative care after non-cardiac thoracic surgery up to now. Therefore, the CT sensitivity for PT or PE, respectively, must be used from other medical fields. Due to this fact, the reliability of these results is uncertain.

Due to the lower accuracy of LUS achieved in several reviewed trials, the mismatch analysis is very important. In several trials, no clinically important pathologies were missed. No clinically relevant PTX (apex-to-cupula >3 cm according to Delphi consensus [51]) was missed by LUS when compared to CXR in the majority of the reviewed trials [17,19,20,22,23,25]. In Smargiassi et al. [18], no PTX was missed by LUS. A diagnostic disagreement between LUS and CXR was clinically irrelevant in most of the cases and will not affect the decision regarding further treatment and patient outcomes. For PTX, the therapeutic agreement between LUS and CXR was from 94% [26] to 97% [19,25]. Galetin et al. have analysed the impact of various patient conditions on LUS sensitivity in PTX detection after thoracic surgery [21]. In 208 patients, the sensitivity of LUS in the diagnosis of PTX was not impaired by age, gender, body mass index, smoking status, severity of chronic obstructive pulmonary disease or by previous ipsilateral operation, irradiation, or thoracotomy. This result confirmed the versatility of LUS when used in postoperative care after non-cardiac thoracic surgery.

The aims of this review were to identify LUS limitations in postoperative care after non-cardiac thoracic surgery, summarize discussed topics and identify possible areas where the evaluation of LUS videos by AI could help to solve these issues. An important limitation for LUS in all performed trials was severe subcutaneous emphysema. Due to the physical nature of the subcutaneous emphysema and its effect on LUS, we do not expect a role of AI here. Wound dressing was not a significant limitation. In Malík et al. [22] and Dzian et al. [23], the missing lung point in case of abolished lung sliding led to high numbers of inconclusive results. As mentioned above, we expect a possible role of AI here. Investigators‘ education and experience are often discussed topics. The subjectivity of LUS findings and the investigator dependency of this modality are also discussed. A visualization of LUS signs detected by AI could possibly help with the objectification of LUS findings, decrease the number of inconclusive LUS results, support the education of beginner investigators and shorten the learning curve. Disadvantages of LUS included the inability to evaluate the chest tube position, limited mediastinum evaluation, and assessment of central lung pathology if it does not reach visceral pleura [18]. In these cases, we do not expect the role of AI due to the physical nature of these limitations or due to pleural and mediastinal anatomy.

### 4.2. AI in LUS Images Evaluation in COVID Patients

LUS has been shown to be an ideal imaging modality for the quick assessment of COVID-19 patients during the COVID-19 pandemic with the ability to predict the clinical course and outcome [43]. A quantification of pulmonary involvement using LUS score in critically ill COVID patients at intensive care unit admission allows predicting the length of intensive care unit stay, the clinical outcome, the need for mechanical ventilation, and death [58]. In this setting, LUS at the bedside could save considerable time when compared to CT (i.e., 80.8 min for each patient) [59]. During the COVID-19 pandemic, AI was increasingly used to assist physicians in LUS videos interpreting and decision making. Multiple studies were performed with different aims [46]. The main tasks were to triage healthy individuals from patients with bacterial or viral pneumonia [60,61], classifying basic LUS signs like A-lines, B-lines, consolidations, pleural effusions, etc. [45], scoring LUS images [62], scoring the severity of the COVID-19 pneumonia [63], etc. During the COVID-19 pandemic, AI was able to achieve high accuracy to differentiate the COVID-19 pneumonia from bacterial and other pneumonia cases and grade the pathology severity [46].

### 4.3. AI in LUS Data Evaluation in Postoperative Care after Non-Cardiac Thoracic Surgery

According to our best knowledge and the performed literature review, there are no studies evaluating the use of AI in the detection of LUS signs in postoperative care after non-cardiac thoracic surgery.

**Figure 3:**
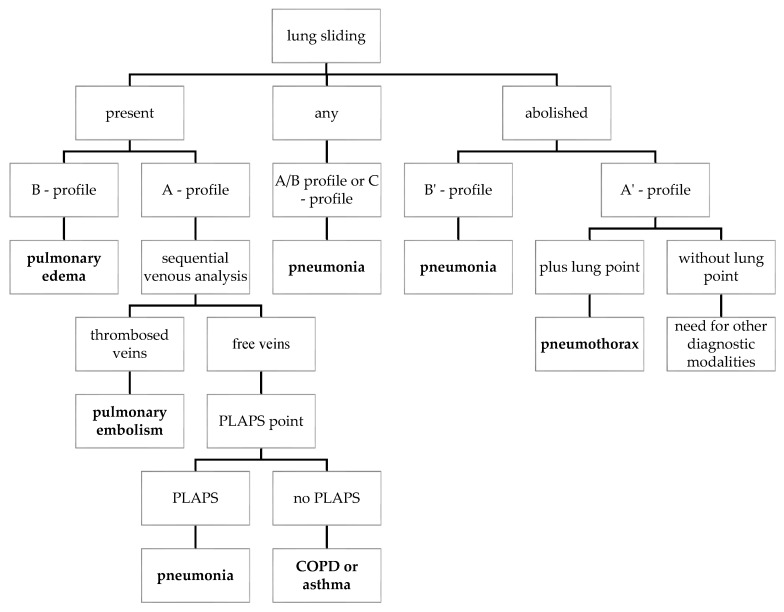
The BLUE protocol [7,8] (A-profile = anterior lung sliding + A-lines; A’-profile = A-profile with abolished lung sliding; B-profile = anterior lung sliding + lung rockets; B’-profile = B-profile with abolished lung sliding; A/B profile = half A profile at one lung and half B-profile at another; C-profile = anterior lung consolidation; PLAPS = PosteroLateral Alveolar and/or Pleural Syndrome; COPD = Chronic Obstructive Pulmonary Disease).

Based on growing evidence of the role of LUS in postoperative care after non-cardiac thoracic surgery [15,16,17,18,19,20,21,22,23,24,25,26] and the growing evidence of the use of AI in the evaluation of LUS images, especially in COVID-19 patients [44,45,46,47], we decided to assess if AI could help to solve the limitations of LUS after thoracic surgery identified in this review. Based on the existing evidence of the use of LUS after thoracic surgery, especially on the results achieved by Galetin et al. [19], we decided to use the BLUE protocol [7]. In our ongoing research, we will step by step train the AI to detect the important LUS signs used in BLUE protocol (Figure 3). The LUS signs relevant in BLUE protocol are: lung sliding [1], B-lines [2,3], A-lines [3], lung point [4], pleural effusion [7] and lung consolidations [6].

In our initial experience, AI was trained to detect the pleural line and to detect the presence or absence of lung sliding on LUS videos from patients after non-cardiac thoracic surgery [48]. LUS videos taken with a linear probe from the upper and lower BLUE points [64] were analyzed using deep learning. A novel deep learning method, automated M-mode classification, was presented. Lung sliding was detected with an accuracy of 89%, sensitivity of 82%, and specificity of 92%. A similar methodology for AI detection of the lung sliding presence and lung sliding absence was used by VanBerlo et al. [65]. The brightness (B) mode videos with lung sliding presence and lung sliding absence were also transformed into the motion (M) mode. A deep neural network was trained to detect seashore sign in case of lung sliding presence and the stratosphere sign in case of lung sliding absence. A total of 2535 LUS videos from 614 patients from various medical fields, by a variety of operators, machines, transducers, and presets, were used for training [65]. The test set included 540 LUS videos from 124 patients, and the model sensitivity, specificity, and an area under the curve were 93.5%; 87.3% and 0.973, respectively [65].

**Figure 4:**
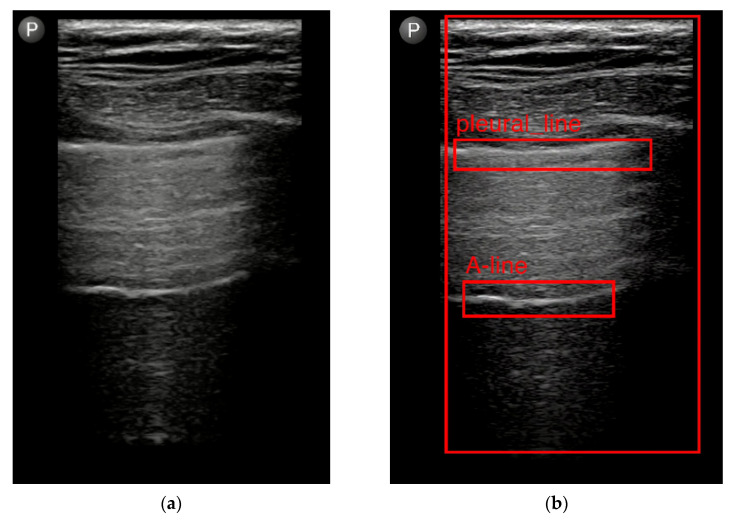
Evaluation of the lung ultrasound video using artificial intelligence: (**a**) Lung ultrasound video from linear probe with A-lines; (**b**) Visualization of anatomical landmarks (pleural line) and ultrasound sign (A-line) after evaluation of the same video as in (**a**) by artificial intelligence using analytical approach [49].

In the next step of our research, AI was trained to detect A-lines and B-lines [49]. Ultrasound videos from patients after various non-cardiac thoracic procedures and LUS videos from a freely available POCUS dataset were used [66]. Analytical computer vision methods were used to detect A-lines [3] and B-lines [2,3]. Using the Fourier transform, LUS images were real-time analyzed and classified with an accuracy above 70%. The method’s applicability for segmentation was also evaluated. Results showed an 89% accuracy rate for B-line and its shortcomings for A-line detection. Increased accuracy for A-lines was achieved using a hybrid solution that combined neural networks training in a pleura of detection and analytical methods. These trials [48,49] represent our preliminary results tested on a small sample size, which is an important limitation. In our ongoing research, we will continue with an evaluation of the rest of the LUS signs relevant in BLUE protocol by AI methods. Finally, a prospective clinical trial on a large patient scale to assess the accuracy of these tools will be conducted. We are working on visualization of the LUS signs detected by AI by highlighting of the LUS sign on the screen (Figure 4). We assume that visualization of the detected LUS signs, especially in real-time conditions, could play a role in the objectification of LUS findings, in improvement of the accuracy of LUS and also in the decision-making process and education of the beginner investigators.

### 4.4. Limitations

The limitation of this review is the inability to perform a meta-analysis of the results from the reviewed trials due to the heterogeneity of the performed trials on several levels, as discussed above in detail.

## 5. Conclusions

LUS as a primary imaging modality performed by an experienced thoracic surgeon combined with clinical examination can safely replace chest X-rays in decision making in postoperative care and chest tube management after non-cardiac thoracic surgery without any negative impact on the patient’s outcome. We presume an important role of artificial intelligence in the implementing of LUS to daily routine in postoperative care after non cardiac thoracic surgery especially by increasing the accuracy, by objectifying lung ultrasound results and helping with the image interpreting, by decreasing the number of the inconclusive results and by beginners’ education with possible shortening of the learning curve. We propose that standardization of the chest tube management and the role of imaging modalities in postoperative care according to evidenced-based medicine principles and the implementation into ERAS protocol could improve the care after non-cardiac thoracic surgery and improve the planning and comparability of the future clinical trials in this field.

## Figures and Tables

**Table 1:** Characteristics and results of the trials evaluating the CXR reduction by LUS in postoperative care and chest tube management after non-cardiac thoracic surgery.

Study	Patients/ Examinations	Study Design	Surgical Procedure	Results			
				PTX Sen/Spe/ PPV/NPV in %	PE Sen/Spe/ PPV/NPV in %	Percentage of Agreement/ Cohen’s Kappa	Percentage of Saved CXR
1. Goudie, 2012 [15]	120/352	LUS when CXR	whole spectrum	21.2/94.7/ 52.7/81.8	83.1/59.3/ 36.1/92.7	NA	NA
2. Chiappetta, 2018 [16]	24/24	LUS and CXR first 48 h after surgery	lung resections (wedge resections, lobectomies) mediastinal tumours resections/biopsy	NA	NA	NA	67% after open procedures, 85% after mini-invasive procedures
3. Patella, 2018 [17]	50/50	LUS and CXR 2 h after chest tube removal	lung resections (wedge resections, lobectomies, segmentectomies)	NA/NA/ 71/100	NA	NA	86%
4. Smargiassi, 2019 [18]	24/24	LUS and CXR first 48 h after surgery	details NA (mini-invasive approach: 16; thoracotomy: 6; robotic thymectomy: 2)	NA	NA	LUS vs. CXR: PTX: 79%/50% PE: 70%/39% LC: 50%/6% SCE: 58%/21% DP: 91%/70%	NA
5. Galetin, 2020 [19]	123/123	LUS and CXR within 1 day after chest tube removal	lung resections and/or chest wall resection	32/85/54/69	NA	Conformity between LUS and CXR-based therapy 97%	NA
				For PTX ≥ 3 cm: 100/82/19/100			
6. Galetin, 2021 [20]	68/68	LUS and CXR on the first day after thoracic surgery and after chest tube removal	lung resection, chest wall resection, decortication	48/81–100/ NA/76	NA	Conformity between LUS and CXR-based therapy 96%	NA
7. Malík, 2021 [22]	297/545	LUS and CXR postoperatively and prior to chest tube removal after its clamping	whole spectrum	1st exam: 59.4/95.9/ 67.9/94.2	1st exam: 44.4/92.6/ 66.7/83.3	LUS vs. CXR 1st exam: PTX 91.3%/58.4% PE 80.5%/41.6%	61.6%
				2nd exam: 50.0/94.8/ 56.5/93.4	2nd exam: 60.9/91.3/ 81.2/79.2	LUS vs. CXR 2nd exam: PTX 89.5%/47.2% PE 79.8%/54.9%	
8. Dzian, 2021 [23]	48/87	LUS and CXR postoperatively and prior to chest tube removal after its clamping	major lung resections (lobectomies/ bilobectomies)	1st exam: 45.5–58.5/91.1–100/77.8–100/72.1–78.7	1st exam: 0–86.2/82.6–88.4/0–33.1/92.5–99	LUS vs. CXR 1st exam: PTX 92.3%/77.5% PE 76.7%/3.6	77%
				2nd exam: 29.7–59.4/79.5–100/50–100/62.2–78.2	2nd exam: 32.6–36.9/68.5–100/88.3–100/12.2–17.8	LUS vs. CXR 2nd exam: PTX 78.8%/39.7% PE 81.1%/61.1%	
9. Messina, 2022 [25]	157/525	Daily LUS and CXR until chest tube removal	major lung resections (lobectomies)	86/100/ 94/94	NA	Conformity between LUS and CXR-based therapy 97%	NA
10. Jakobson, 2022 [26]	80/215	3x LUS and CXR (postoperatively, prior to chest tube removal and 4 h after chest tube removal)	lung resections (anatomical and non-anatomical resections), decortications	NA	NA	LUS/CXR agreement—absolute diagnostic/therapeutic: PTX: 72%/94% PE: 38%/80% LC: 100%/100%	NA

CXR—chest X-ray; DP—diaphragm position; LC—lung consolidations; LUS—lung ultrasound; NA—not available; NPV—negative predictive value; PE—pleural effusion; PPV—positive predictive value; PTX—pneumothorax; SCE—subcutaneous emphysema; Sen—sensitivity; Spe—specificity.

## Data Availability

The data presented in this study are available from the corresponding author on reasonable request.
